# Cell differentiation events in pre-implantation mouse and bovine embryos

**DOI:** 10.1590/1984-3143-AR2021-0054

**Published:** 2022-01-07

**Authors:** Letícia Escobar Carreiro, Gabriel Siqueira dos Santos, Felipe Eduardo Luedke, Marcelo Demarchi Goissis

**Affiliations:** 1 Departamento de Reprodução Animal Faculdade de Medicina Veterinária e Zootecnia Universidade de São Paulo São Paulo SP Brasil Departamento de Reprodução Animal, Faculdade de Medicina Veterinária e Zootecnia, Universidade de São Paulo, São Paulo, SP, Brasil

**Keywords:** early development, embryogenesis, inner cell mass, trophectoderm, blastocyst

## Abstract

Early mammal embryogenesis starts with oocyte fertilization, giving rise to the zygote. The events that the newly formed zygote surpasses are crucial to the embryo developmental success. Shortly after activation of its genome, cells of the embryo segregate into the inner cell mass (ICM) or the trophectoderm (TE). The first will give rise to the embryo while the latter will become the placenta. This first segregation involves cellular and molecular processes that include cell polarity linked to intracellular pathway activation, which will regulate the transcription of trophectoderm-related genes. Then, cells of the ICM undergo the second event of mammalian cell differentiation, which consists of the separation between epiblast (EPI) and hypoblast or primitive endoderm (PrE). This second segregation involves paracrine signaling, leading to differential expression of key genes that will dictate the fate of the cell. Although these processes are described in detail in the mouse, recent studies suggest that the bovine embryo could also be an interesting model for early development, since there are differences to the mouse and similarities with early human embryogenesis. In this review, we gathered the main data available in the literature upon bovine and mouse early development events, suggesting that both models should be analyzed and studied in a complementary way, to better model early events occurring in human development.

## Introduction

In mammals, the formation of the zygote initiates a series of events, known as early embryogenesis. The success of this process depends mainly on the first cell cleavages and the differentiation of the first cell lineages, mechanisms that are essential for the embryo implantation at the uterus and further fetal development (as reviewed by Alberio, 2020).

Present since oogenesis, maternal RNAs and proteins orchestrate the beginning of embryonic development. This maternal material will support basic biosynthetic processes and coordinate the first mitotic divisions. As embryo development progresses, embryonic genome activation (EGA) begins. From this moment on, embryos can develop independently of the maternal genome, until the formation of a blastocyst composed of three distinct cell lineages: the trophectoderm (TE), an outer epithelium made of polarized cells that surrounds a group of nonpolar cells, called the inner cell mass (ICM). The ICM will later differentiate into two distinct cell types, the epiblast (EPI) and the primitive endoderm (PrE) cells (Cockburn and Rossant, 2010; Underhill and Robins, 2016).

The TE is responsible for giving rise to the placental trophoblasts (Underhill and Robins, 2016). On the other hand, the EPI will later differentiate into three intraembryonic tissues, known as endoderm, mesoderm, and ectoderm, which subsequently become the most diverse tissues and organs of the developing fetus (Sheng, 2015). The PrE forms the yolk sac, composed of two extraembryonic cell lineages: visceral and parietal endoderm (Bassalert et al., 2018).

The first cell differentiation of the embryo is widely studied in mice, an animal model that provides the largest source of information about the theme. Although there are some crucial differences in the regulation of early developmental events, some evidence shows that bovine embryos can also be great models for understanding human embryonic development (Chen et al., 2009a; reviewed by Wrenzycki, 2018). Studies indicate that transcription factors such as OCT4 and CDX2, important for the first cell specification, have similar temporal and spatial expression patterns in human (Chen et al., 2009a; Niakan and Eggan, 2013) and bovine (Berg et al., 2011) embryos, while mouse embryos present substantial differences to these species (Niwa et al., 2005; Strumpf et al., 2005; Niakan and Eggan, 2013). In addition, the biological mechanisms that interfere with the regulation of gene expression seem to be different in bovine and mouse embryos (Sakurai et al., 2017; Gerri et al., 2020; Ortega et al., 2020; Anjos et al., 2021).

Considering the importance of the correct specialization of these cell lines for embryo developmental success, this study aims to gather the main data available in the current literature regarding the events of cell differentiation during pre-implantation development, proposing a comparative analysis between the early development of mouse and bovine embryos.

## Developmental dynamics

In mammals, the newly fertilized zygote undergoes symmetrical cell divisions, called cleavages, which is a relatively conserved mechanism between species. In the early stages, the blastomeres are relatively spherical, similar to each other and they become smaller with each cleavage round (Johnson and Ziomek, 1981; Johnson and McConnell, 2004). The events that result in the greatest morphological changes in pre-implantation embryonic development happen during the embryonic compaction, when there is a great increase in intercellular contact followed by the blastocyst formation (Johnson, 2009). Despite the existence of relatively conserved genetic regulatory mechanisms in mammals, the initial development in cattle and mice has relevant differences, mainly in chronological aspects.

In murine embryos, compaction begins at the 8-cell stage, blastomeres flatten their apical surfaces, forming a cluster of cells with indistinct cell boundaries (Lo and Gilula, 1979; Becker and Davies, 1995). At this moment, the apical and basolateral membrane domains are formed, in which the apical domain presents proteins like Ezrin (Louvet et al., 1996) and, the cell adhesion caused by *zonula adheren*t epithelial junction (ZA) characteristic proteins, forms the contact region of basolateral membrane domain between epithelial cells (Aberle et al., 1996).

Furthermore, between 16 and 32-cell stages, Na/K ATPases located at basolateral membranes of mice TE cells transport fluids and ions, forming the blastocoel cavity, filled with blastocoel fluid, characterizing the embryonic cavitation process, which will culminate in the blastocyst formation and an apparent cellular differentiation (Kidder and Watson, 2005).

In vivo produced bovine embryos, however, start compaction near the 32-cell stage, 5 days after insemination, and the cavitation process begins after the 64-cell stage and the early blastocyst contained in average 105 cells (van Soom et al., 1997). Bovine in vitro produced (IVP) embryos initiate cavitation earlier than in vivo ones, after the 32-cell stage. This difference could occur due to observations suggesting that in vivo derived embryos spend more time in compaction (van Soom et al., 1992; van Soom et al., 1997).

**Figure 1:**
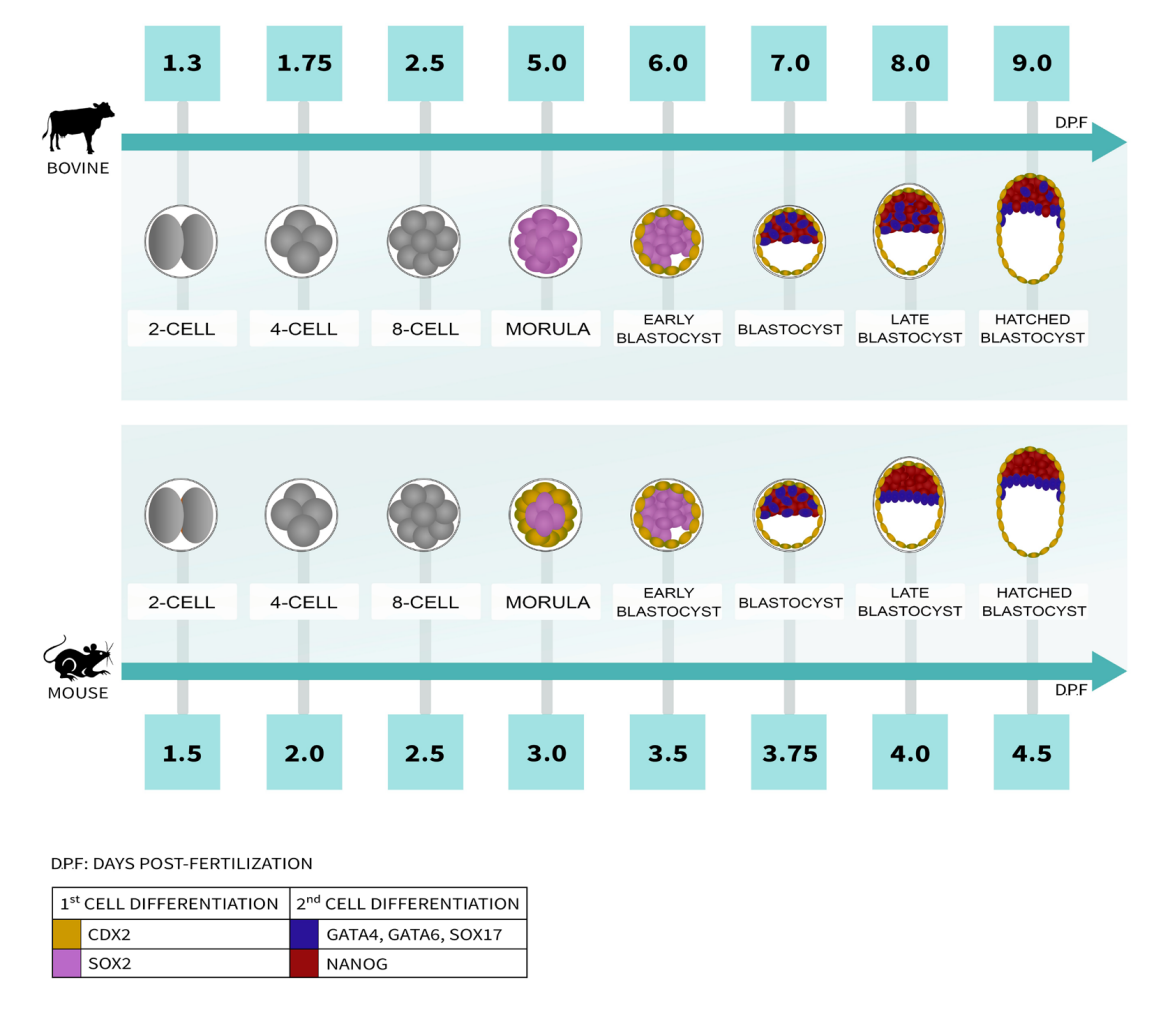
Timeline of morphology and lineage markers expression during early development of bovine and murine embryos. During early development, the main divergences between bovine and mouse embryos occur in the duration of each stage and the occurrence timing of morphological events. For example, compaction gives rise to the morula at 5dpf in bovine and 3dpf in mouse, and the blastocyst formation occurs rapidly in mouse, as soon as 18 hours after morula stage, while bovine blastocyst only forms at 7dpf. Unlike in mouse embryos, bovine morulae do not express trophectoderm marker CDX2 (gold) in outer cells and SOX2 (lilac) is present in all cells. At this point, embryos express epiblast marker NANOG and GATA6 in all cells. After blastocyst formation, NANOG (red) and primitive endoderm markers such as GATA4, GATA6, SOX17 (blue) are mutually exclusive, form a salt-and-pepper expression pattern in the inner cell mass in both bovine and mouse embryos. At the late blastocyst stage in mouse (4.0 dpf), blastomeres expressing either epiblast markers or primitive endoderm markers form different layers while in the hatched blastocyst stage in bovine (9.0 dpf) these two lineages are not still separated into layers. Embryos are represented bidimensionally for ease of understanding.

At this stage of development, the morphological aspect is the most indicative factor on embryo quality for embryo transfer (Watson et al., 1999). Although the developmental stages present a very similar morphology in bovine and mouse embryos, there are divergences in the duration of each stage and the occurrence timing of remarkable morphological events, such as the compaction and formation of the blastocoel (Figure 1). Still, there are differences in expression pattern and function of proteins related to TE cells specification (such as CDX2 and YAP) and ICM (OCT4, SOX2, NANOG, GATA4, GATA6, and SOX17) (Yamanaka et al., 2006; Wicklow et al., 2014; Wei et al., 2017; Sharma and Madan, 2020). The genetic regulation mechanisms, protein expression profiles, and cell signaling pathways related to cell differentiation events are going to be presented next.

## First cell differentiation event

In mammals, the first cleavages will give rise to an embryo composed of totipotent blastomeres, with full differentiation potential in all cell lineages (Fleming and Johnson, 1988). During development, the first cell differentiation leads to the formation of TE epithelial cells or ICM cells. In the post-implantation stages, the TE will give rise to the extra-embryonic ectoderm and the placental trophoblasts, while the ICM will be responsible for developing the embryo itself and the extra-embryonic endoderm (Strumpf et al., 2005).

Two models that seek to explain the mechanisms involved in the first cell differentiation in the mouse are currently accepted. The first one, called the inside-outside model (Tarkowski and Wróblewska, 1967), proposes that the cell position in the embryo is the main factor that will define the cell type fate. The second model, which does not exclude the first one, argues that cell polarization defines cell lineage (Johnson and Ziomek, 1981). According to this model, polarization and the beginning of asymmetric cell divisions are crucial events for cell specifying process initiation (Graham and Deussen, 1978; Johnson and Ziomek, 1981; Fleming, 1987).

Despite these studies, there is no consensus on the exact moment when blastomeres determine their cell lineage fate (Posfai et al., 2017). In mice, before these two cell lines segregate, evidence shows that gene expression pattern starts to change between internal and external cells at the 8-cell stage. As a result, genes initially expressed in all blastomeres start to have their expressions regulated according to the cell location (Pesce and Scholer, 2001).

Embryonic compaction promotes an increase in cell-cell interactions and cell adhesion proteins, resulting in cell polarization (Chen and Zhang, 2013). The appearance of polarity-related proteins, such as PARD3, PARD6, and PKCs, was already reported in the mouse morula stage (Plusa et al., 2005). The organization of these proteins during compaction leads to an apical domain formation. Once cells express two distinct intercellular junction complexes, tight and adherent, an apical region and a basolateral region establish cellular polarity (Plusa et al., 2005; Vinot et al., 2005; Ralston and Rossant, 2008).

**Figure 2:**
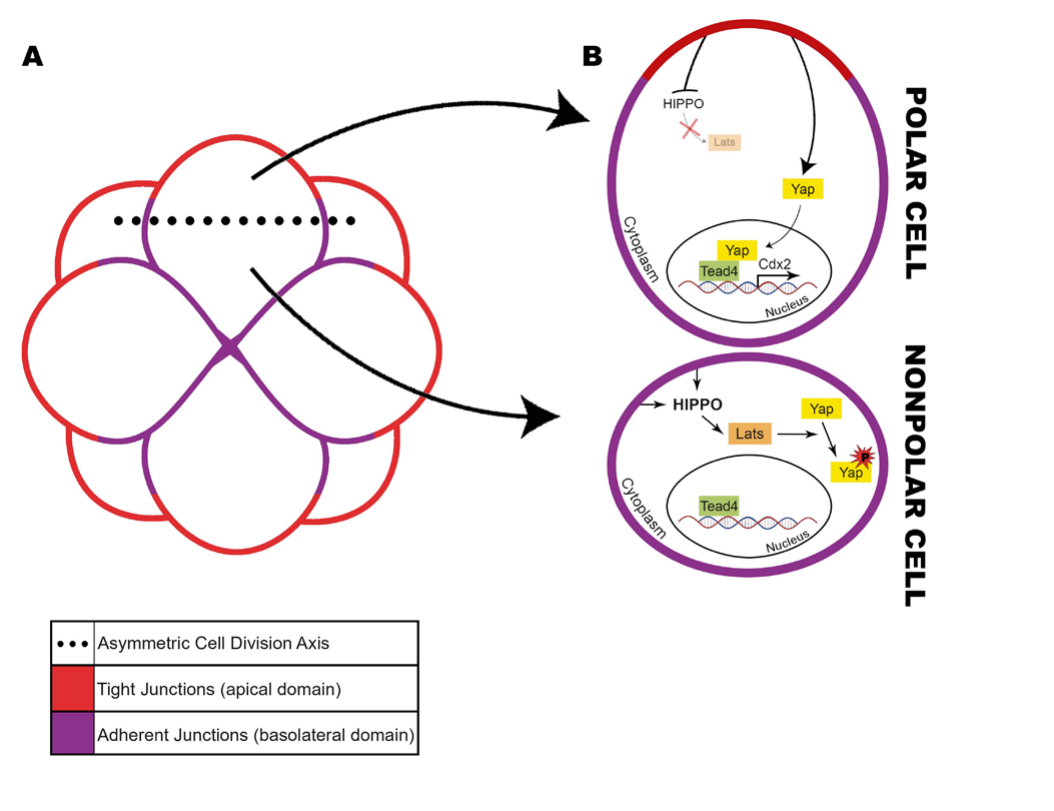
Influence of cell polarity and Hippo signaling pathway in TE and ICM cell differentiation in mouse embryos. (A) At the 8-cell stage, the tight junctions (red) and the adherent junctions (purple) establish a polarization pattern in the embryo. The asymmetric cell division axis (dotted line) is responsible for generating two types of daughter cells; (B) The first, an externally located polar cell, which will inherit both apical and basolateral domains, and the second one, internally positioned, which will acquire only the basolateral domain proteins, distributed homogeneously, becoming then nonpolar cells. The apical domain blocks the Hippo pathway activation in polar cells, allowing YAP1 translocation into the nucleus, and then resulting in *Cdx2* transcription. With the absence of tight junctions, Hippo pathway is activated at nonpolar cells.

At this stage of development, asymmetric cell divisions are responsible for generating two types of daughter cells: an externally located polar cell, which will inherit both apical and basolateral domains, and an internally positioned cell, which will acquire only the basolateral domain proteins, distributed homogeneously, becoming then nonpolar cells (Figure 2A). In bovine embryos, recent research reported a similar cellular polarity pattern to murine embryos, essential for the beginning of TE specification; however, polarization inhibition did not show as drastic effects as in mouse embryos (Gerri et al., 2020) and another study showed that inhibition of apical domain formation using blebbistatin did not impair bovine blastocyst formation (Anjos et al., 2021), suggesting that polarization is not the only process controlling this differentiation in bovine embryos.

In the mouse, cell division asymmetry is also responsible for generating blastomeres with different cell contractility. Polar cells maintain low contractility in the apical domain, while nonpolar cells, which have an increase in cell contractility, compete for internal positions (Maître et al., 2016). Studies indicate that differences in the organization of cytoskeleton proteins occur before the spatial organization of these cells, contributing to cell differentiation. Mouse blastomeres with apical domain show an increase in cell surface keratins expression, which prevents the internalization of these cells and consequently precedes the expression of TE markers (Lim et al., 2020).

Polarity establishment and the asymmetric cell divisions influence Hippo pathway activity, which behaves differently in polar and nonpolar cells, controlling cellular fate (Yamanaka et al., 2006; Nishioka et al., 2009) as externally positioned cells acquire an apical domain, which influences the Hippo pathway, giving rise to TE in mouse embryos (Korotkevich et al., 2017). The Hippo pathway activation in the internal nonpolar cells causes LATS1/2 serine/threonine kinases to promote YAP1 protein phosphorylation, which prevents its transport to the nucleus. Once phosphorylated, YAP is not able to act as a transcription cofactor together with TEAD4. In the polar apical cells, the Hippo pathway remains inactive and YAP1 is translocated to the nucleus, where it will function as a TEAD4 co-activator (Figure 2B). TEAD4 is a transcription factor member of the TEA domain family, essential for the maintenance and/or positive regulation of *Cdx2* in mouse, which expression directly relates to further development of TE (Yagi et al., 2007; Nishioka et al., 2008; Nishioka et al., 2009; Misra and Irvine, 2018).

*Tead4* deletion in mouse embryos increased the expression of specific ICM factors, such as *Oct4, Nanog*, and *Sox2,* suggesting that *Tead4* is not essential for ICM formation (Nishioka et al., 2008). Bovine embryos have low *TEAD4* mRNA levels in the early stages of development, increasing their expression from the 16-cell stage. During the 8 to 16-cell stage, TEAD4 is detected in the cytoplasm, while in the morula and blastocyst it is located in the nucleus of most blastomeres. Bovine embryos in which *TEAD4* was knocked down using small interfering RNA (siRNA) present blastocyst formation and normal TE cell differentiation and expansion. Unlike in mice, *TEAD4* knockdown did not influence the gene expression of *OCT4*, *NANOG, CDX2*, and *GATA3*, suggesting that TEAD4 is not essential for TE formation in the bovine embryo (Sakurai et al., 2017).

Deletion of *Yap1* and *Wwtr1* (TAZ), a functional homolog of YAP1, led to the death of mouse embryos before reaching the morula stage. By contrast, overexpression of *Yap1* and *Taz* promotes the expression of *Cdx2*, confirming the hypothesis that YAP1 together with TAZ and TEAD4 regulates the expression of *Cdx2* (Nishioka et al., 2009).

Recently, differences in YAP1 and TAZ patterns were reported in bovine embryos, suggesting that in these animals, Hippo signaling may be established by different mechanisms. Despite the pattern shown in murine embryos, phosphorylated YAP1 is found both in the nucleus and in the cytoplasm of bovine embryos, from 2-cell to the 16-cell stage, becoming mostly nuclear from the morula onwards (Sharma and Madan, 2020). The TAZ coactivator exhibited nuclear localization in the external cells of the blastocyst, whereas, in mice, TAZ localization becomes exclusively nuclear from the morula stage onwards (Sharma and Madan, 2020).

The Hippo signaling pathway is also related to glucose metabolism, which in turn may be related to this first cell differentiation event. In the mouse, it was first demonstrated that transcription factor TEAD4 was not necessary for the formation of the blastocoel when embryos were cultured in conditions with lower concentrations of oxygen. However, *Tead4* knockout embryos cultured in the absence of glucose did not initiate blastocoel formation, unlike wild-type embryos, suggesting that the role of TEAD4 during pre-implantation development is to establish the essential energy homeostasis for the transition from the morula to blastocyst (Kaneko and DePamphilis, 2013).

The relationship between glucose metabolism and blastocyst formation is known for some time. Shortly before the formation of the blastocoel, the mouse embryo abruptly changes its energy sources from pyruvate and lactate to glucose, concomitantly with a 2.7-fold increase in oxygen consumption (Gardner, 1998; Johnson et al., 2003). Brown and Whittingham (1991) established a specific glucose requirement during the transition from morula to blastocyst, and unlike the early stages of mouse embryonic development in which lactate and pyruvate meet the needs of the embryo; glucose is required during the first cell differentiation.

In mice, when glucose was not available in the medium, a developmental block occurred in the eight-cell stage and there was no expression of the transcription factor *Cdx2*, ensuing just the expression of ICM-related transcription factors (Chi et al., 2020). It was also shown that the expression of *Cdx2* was influenced by the modulation of the hexosamine biosynthetic pathway and pentose phosphate pathway (Chi et al., 2020).

These pathways would be involved in the O-glycosylation of YAP1, allowing nuclear translocation of YAP1, directly interfering in the first cell differentiation process and playing a decisive role in the specification of the trophectoderm (Chi et al., 2020). More specifically, pharmacological modulation of the O-GlcNAcylation process in pre-implantation stages proved to be important for future differentiation of trophoblast cells, interfering with the expression of transcription factors such as CDX2 and GATA3 (Ruane et al., 2020).

It is known that the *Cdx2* transcription factor is an important regulator and is abundantly present in TE cells (Beck et al., 1995). In mouse embryos, since the 8-cell stage, Cdx2 mRNA traces are identified, and Cdx2 expression is observed in the morula stage (approximately from 16-cells) in the nucleus and the cytoplasm of outer cells. At the expanded blastocyst stage, Cdx2 is restricted to the nucleus of these cells (Strumpf et al., 2005; Ralston and Rossant, 2005). In bovine embryos, CDX2 is present only at the blastocyst stage (Madeja et al., 2013; Goissis and Cibelli, 2014b). The deletion of *Cdx2* in mouse and bovine embryos influences the epithelium integrity, which becomes more permeable with CDX2 silencing, but does not impair TE or blastocyst formation, reinforcing that TE differentiation is regulated by factors or pathways hierarchically superior to CDX2 (Strumpf et al., 2005; Wu et al., 2010; Goissis and Cibelli, 2014b).

In the internal apolar cells, transcription factors related to the ICM pluripotency maintenance such as *Oct4* (also known as Pou5f1), *Sox2*, and *Nanog*, are initially expressed (Schöler et al., 1990; Niwa et al., 2000; Chambers et al., 2003; Nishioka et al., 2008). In the mouse morula early stages, blastomeres have both Cdx2 and Oct4 expression, but gradually these factors develop a mutual exclusive regulation. *Cdx2* or *Oct4* exclusive expression is essential for the establishment of TE and ICM strains, respectively (Niwa et al., 2005; Ralston and Rossant, 2005; Strumpf et al., 2005; Guo et al., 2010). This same mutual suppression mechanism has also been observed between *Nanog* and *Cdx2*, indicating *Cdx2* role in restricting these transcripts to the ICM (Chen et al., 2009b).

In cattle, however, OCT4 is detected in TE and ICM cells, even in the blastocyst stage (Kirchhof et al., 2000; Kuijk et al., 2008), suggesting that in these animals, OCT4 may be involved in other regulatory mechanisms. There is evidence that, unlike what has already been reported in mice, CDX2 does not negatively regulate OCT4 transcription in bovine embryos, due to OCT4 gene promoter region differences in cattle and other species, including human, when compared to mouse (Berg et al., 2011). Still, other studies have demonstrated that CDX2 silencing does not affect OCT4 detection (Goissis and Cibelli, 2014b; Sakurai et al., 2017).

Oct4 knockout murine embryos are able to form blastocysts, although these embryos ICM is not pluripotent, and the cells differentiate into extraembryonic trophoblastic strains only (Nichols et al., 1998), suggesting a requirement for OCT4 in order to achieve ICM development (Niwa et al., 2000). *OCT4* deletion using CRISPR/Cas9 in bovine embryos demonstrated that blastocyst initial formation depends on OCT4 expression since knockout embryos were blocked at the morula stage, prior to the first cell specification, similar to human embryos (Fogarty et al., 2017; Daigneault et al., 2018). Studies have also indicated that possibly OCT4 has a regulatory role in cattle TE differentiation, probably through CDX2 regulation (Daigneault et al., 2018).

Also necessary for pluripotency maintenance (Avilion et al., 2003), SOX2 expression is ICM-restricted at murine blastocysts (Guo et al., 2010). In mice, maternal SOX2 is located in the nucleus already in 2-cell embryos, while *Sox2* zygotic transcription begins lately, at the 16-cell stage, in ICM precursor cells (Avilion et al., 2003; Guo et al., 2010). Despite the early NANOG, OCT4, and CDX2 co-localization pattern, there is no evidence of SOX2 expression in external CDX2-positive cells, being the first exclusive marker of ICM-precursors. Studies reveal that SOX2 is restricted to the ICM by CDX2-independent mechanisms but it requires Tead4 and Lats2 (Wicklow et al., 2014). Maternal YAP1 and TAZ and zygotic TEAD4 directly prevent SOX2 expression in the early stages of development, a mechanism that ceases in cells that have positioned themselves internally, by the 16-cell stage (Frum et al., 2019). Interestingly, mouse blastomeres showing SOX2 long-term binding to DNA, at the stage of 4-cells, were predisposed to differentiate into ICM (White et al., 2016).

The silencing of maternal and zygotic SOX2 protein impairs mouse blastocyst formation. Most of these embryos have their development stagnated at the morula stage, failing to cavitate. In these modified embryos, the lack of SOX2 does not affect *Oct4* and *Nanog* expression but results in a large reduction in TE transcripts, such as TEAD4, CDX2, Eomes, and YAP, but not GATA3 (Keramari et al., 2010). Other studies suggest that Sox2 knockout does not prevent blastocyst formation, but ICM cells differentiate into TE lineage or extraembryonic endoderm (Avilion et al., 2003).

In bovine embryos, expression of SOX2 is observed in all cells from the 16-cell stage to the morula stage, while restricted to the ICM in the blastocyst. The knockdown of SOX2 in these embryos reduced NANOG expression in blastocysts, suggesting a regulatory relationship between these transcripts. Unlike murine embryos, OCT4 levels were not compromised, suggesting that SOX2 and OCT4 may not establish a direct regulatory relationship in cattle (Goissis and Cibelli, 2014a; Xie et al., 2010).

## Second cell differentiation event

Initially, it was believed that the decisive factor for cell differentiation within the ICM of murine embryos would be the cell location. The cells near the blastocoel would differentiate into PrE, while the innermost cells of the ICM would give rise to EPI (Rossant, 1975). This hypothesis, however, was challenged by demonstration of differential expression of transcription factors in ICM cells, such as *Nanog* and *Gata6*, which are essential for the establishment of a blastocyst composed of three cell lineages (Mitsui et al., 2003; Chazaud et al., 2006; Plusa et al., 2008).

In the late blastocyst, EPI cells are characterized by the expression of pluripotency factors such as *Nanog* (Mitsui et al., 2003; Chambers et al., 2003) and *Sox2* (Avilion et al., 2003), while PrE cells have a sequential expression activation of *Gata6*, *Gata4* (Fujikura et al., 2002), *Sox17* and *Sox7* (Artus et al., 2011). In mice, NANOG (Mitsui et al., 2003) and GATA6 (Fujikura et al., 2002) are co-expressed in all cells from the 8-cell stage to the blastocyst. At the blastocyst stage, these transcription factors become mutually exclusive in ICM cells, characterizing the “salt-and-pepper pattern”, before the establishment of EPI and PrE lineages (Rossant et al., 2003; Chazaud et al., 2006; Plusa et al., 2008; Guo et al., 2010).

In cattle, NANOG expression in the ICM begins at the early blastocyst stage, increasing its expression at days 7 and 8 of development (Kuijk et al., 2012), although there is some evidence of the onset of NANOG expression at the 8-cell stage (Canizo et al., 2019). GATA6 expression was detected in all embryo cells from the morula stage and its expression was restricted to ICM only after developmental day 8, already at the blastocyst stage. As in murine embryos, blastomeres initially express both NANOG and GATA6, and they progressively become restricted to EPI and PrE precursor cells (Kuijk et al., 2012).

Knockout of *Nanog* in mouse embryos did not alter GATA6 expression, indicating that the initiation of PrE differentiation is independent of *Nanog* (Frankenberg et al., 2011). Nanog knockdown in embryos using siRNA also have shown GATA6-positive across the ICM, however, these cells failed to activate the PrE differentiation program, evidenced by the absence of Gata4 and Sox17 expression (Frankenberg et al., 2011). Contrary to in the mouse, knockout of *NANOG* in bovine embryos reduced *GATA6* expression (Ortega et al., 2020), suggesting different PrE differentiation dynamics in the bovine embryo.

**Figure 3:**
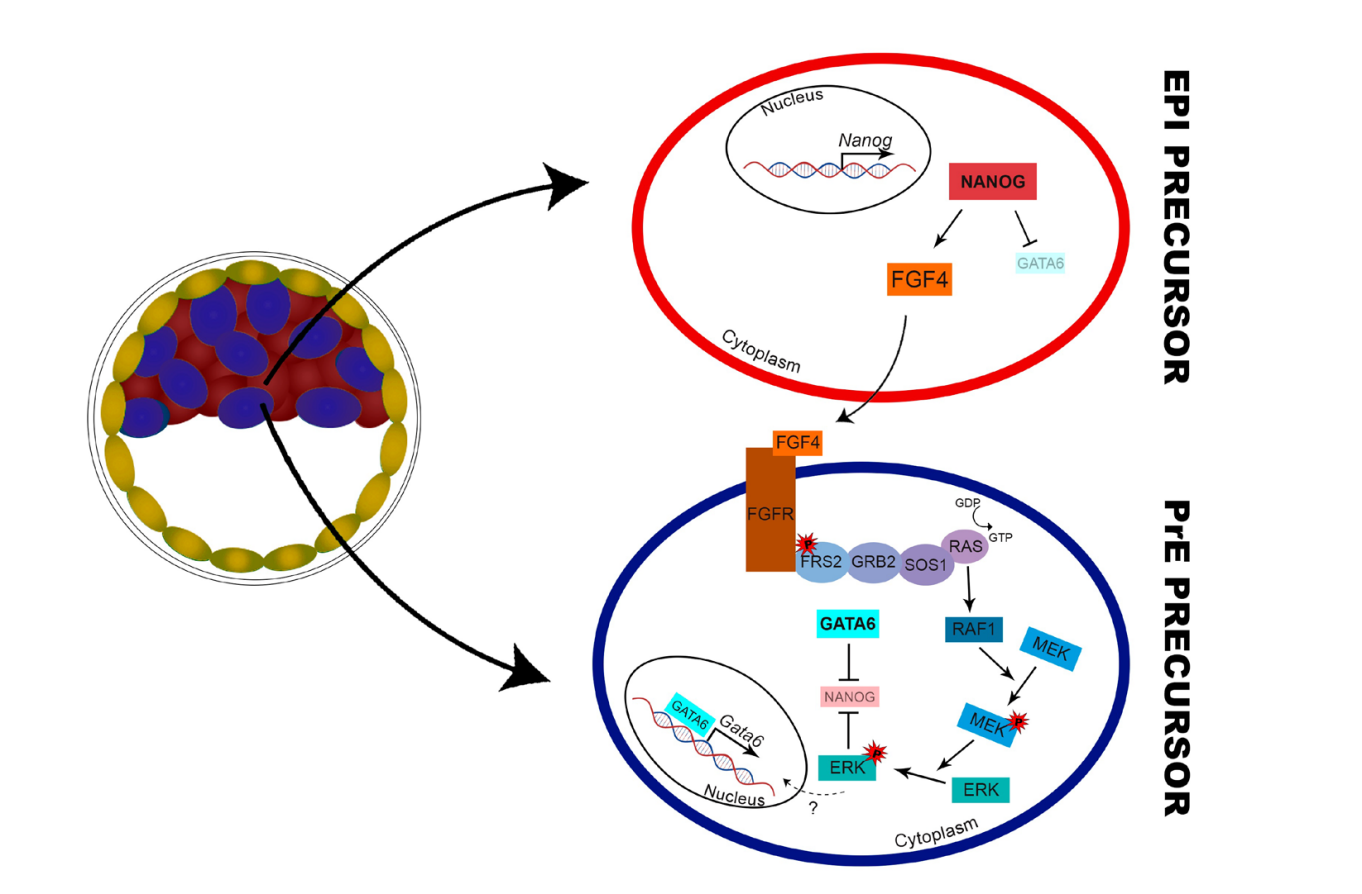
FGF/ERK role at EPI and PrE cell specification in mouse embryos. In the early blastocyst stage, NANOG expression directly regulates *Fgf4* transcription, while it downregulates GATA6 expression in EPI precursor cells (red). FGF4, in a paracrine manner, binds to the receptors (FGFRs) in neighboring cells, activating the ERK cascade in PrE precursor cells (blue), leading to GATA6 expression and further inhibition of NANOG.

One of the signaling pathways that regulate the second differentiation is the FGF/ERK pathway, activated by FGF4 binding to its receptors (FGFRs) (Yamanaka et al., 2010; Kang et al., 2013; Molotkov et al., 2017; Soszynska et al., 2019). In mouse embryos, *Fgf4* expression could be observed in EPI precursor cells, directly regulated by NANOG (Frankenberg et al., 2011) and, in the neighboring cells, FGF4 binds to its receptors, activating the PrE differentiation program (Yamanaka et al., 2010; Frankenberg et al., 2011) (Figure 3).

Mouse embryos with *Fgf4* deletion showed NANOG and GATA6 labeling in most ICM cells, from the morula stage to early blastocysts, suggesting that the initial expression of these proteins does not depend on the signaling triggered by FGF4. However, with further development of blastocysts, these embryos formed a predominantly NANOG-positive ICM, suggesting that FGF4 is necessary for the maintenance of GATA6 expression in ICM. The GATA4 and SOX17 expression was not detected in these Fgf4^-/-^ embryos, indicating the need for an active PrE cell differentiation program (Kang et al., 2013).

Bovine embryos treated with exogenous FGF4 formed blastocysts with an ICM composed exclusively of GATA6-positive cells, demonstrating that FGF4 presence can induce gene expression pattern of the PrE precursors and block differentiation into EPI cells (Kuijk et al., 2012). Studies have also shown that exogenous FGF2 was also able to induce the formation and proliferation of PrE in bovine cells in vitro, indicating that other resources can be used to trigger the activation of the cell differentiation program in these animals (Yang et al., 2011).

Studies indicate that the activity of the FGF/ERK pathway depends on the types of FGFR expressed in the cells. Studies in mice embryos have shown that both EPI and PrE precursors present *Fgfr1* expression, and *Fgfr2* is preferentially expressed in PrE precursor cells only. *Fgfr1* or *Fgfr2* knockouts demonstrated that FGFR1 has a fundamental role in PrE differentiation, while FGFR2 played a secondary role (Molotkov et al., 2017). There are reports describing FGFR1 and FGFR3 expression in the ICM of bovine embryos, but without functional information (Ozawa et al., 2013). The location of bovine FGFR2 is controversial, as studies have indicated its presence restricted to TE cells (Ozawa et al., 2013), but ICM expression has also been described (Akizawa et al., 2016) and mainly in PrE cells (Negrón-Pérez and Hansen, 2018).

FGFRs activation causes the recruitment of adapter proteins that will connect FGF signaling and the ERK pathway (Wang et al., 2004; Soszynska et al., 2019). ERKs activation negatively regulates NANOG, since the ERK1-mediated NANOG phosphorylation subsequently caused NANOG degradation (Kim et al., 2014), generating an imbalance in the early coexpression pattern of NANOG and GATA6 and contributing to cellular specification into PrE (Plusa et al., 2008; Guo et al., 2010). Manipulation of FGF/ERK downstream pathway elements has evidenced its importance for the initial development success. The deletion of *Grb2* in mouse embryos did not impair ICM compaction, but inhibited Gata6 and the expression of other PrE markers, resulting in the absence of PrE formation. However, these embryos maintained *Nanog* expression in all ICM cells, which developed into EPI, suggesting the role of the FGF/ERK pathway in PrE development (Chazaud et al., 2006).

Treatment of mouse embryos with FGFR or MEK inhibitors caused the same phenotype observed in blastocysts presenting *Grb2* or *Gata6* deletion. However, when the treatment was interrupted at the 16-cell stage, there were no changes observed in the expression pattern of ICM transcription factors, suggesting that the FGF/ERK signaling pathway activation occurs in the early blastocyst stages (Yamanaka et al., 2010).

Bovine embryos treated with MEK inhibitors had a significant decrease in TE and ICM cells numbers (Canizo et al., 2019), as well as increased expression of NANOG and FGF4, and decreased expression of GATA4 and SOX17, at the blastocyst stage, indicating the direct ERK pathway influence at the ICM differentiation process. However, GATA6 labeling was observed in some blastomeres, showing that some cells maintain PrE differentiation program regardless of ERK activation (Kuijk et al., 2012; Harris et al., 2013; McLean et al., 2014; Negrón-Pérez and Hansen, 2018; Canizo et al., 2019).

This differentiation process is essentially GATA6-dependent in mice since *Gata6* knockout embryos did not form PrE lineage, expressing NANOG in all ICM cells (Schrode et al., 2014). Late expression of transcription factors related to PrE, such as SOX17 and GATA4, seemed dependent on Gata6 and Fgf4-activated signaling (Bessonnard et al., 2014). Still, mouse wild type and *Gata6* knockout embryos treated with exogenous FGF4 in the 8 cell stage, did not express NANOG, showing that the FGF/ERK pathway can inhibit NANOG independently of *Gata6*. However, when they were treated in later stages, as in the early blastocyst, NANOG expression was maintained (Bessonnard et al., 2014). Currently, no studies downregulated or ablated GATA6 in bovine embryos.

## Conclusion

The main knowledge of early embryonic development in mammals derived mostly from studies in mouse models. Research in bovine embryos shows that the biological processes involved in early cell differentiation are not as conserved (summarized in Table 1), for example, the timing and molecular control of CDX2 expression, or the molecular control of genes related to the second cell differentiation. There are still significant literature information gaps about pre-implantation cell differentiation events in humans and previously presented evidence indicates that human embryos have some patterns and regulatory mechanisms that are more similar to bovine embryos. Thus, we suggest the that bovine and mouse models should be studied in parallel for further understanding of the biological mechanisms involved in early embryonic morphological events.

**Table 1:** Summary of key events and biological mechanisms ocurring in mouse and bovine embryos. EPI: Epiblast; FGFR: Fibroblast Growth Factor Receptor; ICM: Inner Cell Mass; PrE: primitive endoderm; TE: Trophectoderm.

**Observation**	**Murine embryo**	**Bovine embryo**	**References**
Stage in which embryo compaction occurs	8-cell	32-cell	(Lo and Gilula, 1979; Becker and Davies, 1995; van Soom et al., 1997)
Stage in which embryo cavitation occurs	Between 16 and 32-cell	Between 32 and 64-cell	(van Soom et al., 1992; van Soom et al., 1997; Kidder and Watson, 2005)
Is *TEAD4* essential for TE formation?	Yes	No	(Kaneko and Depamphilis, 2013; Sakurai et al., 2017)
Does *CDX2* have any function in epithelium integrity?	Yes	Yes	(Strumpf et al., 2005; Goissis and Cibelli, 2014b)
Does *CDX2* delection impair TE or blastocyst formation?	No	No	(Strumpf et al., 2005; Wu et al., 2010; Goissis and Cibelli, 2014b)
Where is *OCT4* detected after first differentiation?	In ICM cells	In TE and ICM cells	(Ralston and Rossant, 2005; Strumpf et al., 2005; Kuijk et al., 2008)
Does *CDX2* negatively regulate *OCT4* transcription?	Yes	No	(Berg et al., 2011; Goissis and Cibelli, 2014b; Sakurai et al., 2017)
*OCT4* knockout leads to TE differentiation	Yes	No (morula block)	(Nichols et al., 1998; Daigneault et al., 2018)
Do *SOX2* and *OCT4* establish a direct regulatory relationship ?	Yes	No	(Goissis and Cibelli, 2014a; Xie et al., 2010)
*NANOG* knockout in ICM differentiation	Maintain initial *GATA6* expression but impairs both EPI and PrE	Reduces *GATA6* expression and impairs epiblast differentiation	(Frankenberg et al., 2011; Ortega et al., 2020)
Is FGF4 necessary for the maintenance of *GATA6* expression in ICM?	Yes	Yes	(Kuijk et al., 2012; Kang et al., 2013)
FGFR inhibition effects	Eliminate GATA6 and increase NANOG+ cells in ICM	Doesn't alter the distribution of NANOG or GATA6+ cells	(Yamanaka et al., 2010; Kujik et al., 2012)
MEK inhibition effects	Eliminate GATA6+ and increase *NANOG*+ cells in ICM	Reduce *GATA6* and enhance NANOG+ cells in ICM	(Yamanaka et al., 2010; Kujik et al., 2012; Canizo et al., 2019)
Types of FGFR identified in ICM	Fgfr1 and Fgfr 2	Fgfr1 and Fgfr3. Possibly Fgfr 2	(Ozawa et al., 2013; Akizawa et al., 2016; Molotkov et al., 2017; Negrón-Pérez and Hansen, 2018)
